# Supplementary material for: Design and fault diagnosis of DCS sintering furnace’s temperature control system for edge computing
Source: PLoS One. 2021 Jul 6;16(7):e0253246. doi: 10.1371/journal.pone.0253246 (PMC8259965; doi:10.1371/journal.pone.0253246)
Supplement: Attachment — Submitted filename: comments_majorevision.docx [file pone.0253246.s002.docx]

Reviewer #1:

1. Gap in the article is not presented properly by the authors.

Response: Thanks for reviewing this article. A “Related Work” section has been supplemented to present the research gaps.
2. Author failed to highlight contributions in this article
Response: Contributions have been highlighted in the introduction section.
3. The limitations of the existing works which prompted the authors to carry out the current research can be discussed in introduction.
Response: A “Related Work” section has been supplemented to discuss the limitations of the existing works which prompted the current research.
4. Related works section can be summarized as a table.
Response: A “Related Work” section has been supplemented.
5. Figures are not clear. Unable to see the text in figures. Redraw the figures.
Response: The figures have been redrawn.
6. How is the paper different from existing works? What gaps they identified in existing works? How the proposed approach solves the research/scientific problems identified in the existing works?
Response: In the supplemented “Related Work” section, the differences between the paper and existing works have been explained; besides, the contributions of this paper have been highlighted in the conclusion section.
7. Authors should tabulate the values obtained for the performance measures used to evaluate their contribution.

Response: The system test results are indicators for evaluating the contribution of the paper, which have been shown through figures.

Reviewer #2:

1. The authors have to carefully proofread the paper to improve it grammatically.
Response: Thanks for your comment. The paper has been proofread.
2. WHat are the limitations of the existing works?
Response: A “Related Work” section has been supplemented to discuss the limitations of the existing works which prompted the current research.
3. List out the main contributions of the current work.
Response: The contributions of this paper have been highlighted in the conclusion section.
4. Compare the current work with recent state-of-the-art.
Response: The current work has been compared with recent state-of-the-art.
5. Discuss how machine learning can be useful for fault diagnosis. The authors can refer these articles"An adaptive multi-layer botnet detection technique using machine learning classifiers, A novel ensemble of hybrid intrusion detection system for detecting internet of things attacks" which use ML for different applications.
Response: This article has been cited (reference [33]).
6. Present a detailed analysis on the results obtained.

Response: A detailed analysis on the results obtained has been presented.

Reviewer #3:

1. Abstract add the results achieved
Response: Thanks for reviewing this paper. The results achieved have been added in the abstract.
2. In introduction add the contributions
Response: Contributions have been added in the introduction section.
3. Add the latest references
Response: Latest references have been cited in this revision.
4. figures quality has to be improved
Response: Figures have been redrawn.
5. The authors can cite the following references
Fusion of Federated Learning and Industrial Internet of Things: A Survey
A survey on blockchain for big data: Approaches, opportunities, and future directions

Response: As you have suggested, these works have been cited (reference [3] and [5]).
